# A Microfluidic 3D Endothelium-on-a-Chip Model to Study Transendothelial Migration of T Cells in Health and Disease

**DOI:** 10.3390/ijms22158234

**Published:** 2021-07-30

**Authors:** Luuk de Haan, Johnny Suijker, Ruthger van Roey, Nina Berges, Elissaveta Petrova, Karla Queiroz, Wouter Strijker, Thomas Olivier, Oliver Poeschke, Sakshi Garg, Lenie J. van den Broek

**Affiliations:** 1 Mimetas BV, de Limes 7, 2342 DH Oegstgeest, The Netherlands; l.dehaan@mimetas.com (L.d.H.); j.suijker@mimetas.com (J.S.); Ruthgervr@live.nl (R.v.R.); k.queiroz@mimetas.com (K.Q.); w.strijker@mimetas.com (W.S.); t.olivier@mimetas.com (T.O.); 2 Merck Healthcare KGaA, Frankfurter Str. 250, 64293 Darmstadt, Germany; nina.berges@merckgroup.com (N.B.); elissaveta.petrova@merckgroup.com (E.P.); Oliver.Poeschke@merckgroup.com (O.P.); sakshi.garg@merckgroup.com (S.G.)

**Keywords:** T cell, microfluidic, high-throughput, transendothelial migration, extravasation, inflammation, immuno-oncology, in vitro, organ-on-a-chip

## Abstract

The recruitment of T cells is a crucial component in the inflammatory cascade of the body. The process involves the transport of T cells through the vascular system and their stable arrest to vessel walls at the site of inflammation, followed by extravasation and subsequent infiltration into tissue. Here, we describe an assay to study 3D T cell dynamics under flow in real time using a high-throughput, artificial membrane-free microfluidic platform that allows unimpeded extravasation of T cells. We show that primary human T cells adhere to endothelial vessel walls upon perfusion of microvessels and can be stimulated to undergo transendothelial migration (TEM) by TNFα-mediated vascular inflammation and the presence of CXCL12 gradients or ECM-embedded melanoma cells. Notably, migratory behavior was found to differ depending on T cell activation states. The assay is unique in its comprehensiveness for modelling T cell trafficking, arrest, extravasation and migration, all in one system, combined with its throughput, quality of imaging and ease of use. We envision routine use of this assay to study immunological processes and expect it to spur research in the fields of immunological disorders, immuno-oncology and the development of novel immunotherapeutics.

## 1. Introduction

The immune system is a complex system that provides protection against potential threats to the integrity of the human body [1]. It consists of various cell types with immensely diverse and highly specific receptors to recognize these threats and distinguish self from non-self. In immune function disorders, such as auto-immune diseases [2] or cancer [3], the immune system fails to distinguish healthy cells from potential threats, either targeting healthy, functional self-cells or failing to detect and kill proliferating tumor cells. One important step in the immunological cascade in both health and disease is the recruitment of T cells towards the site of inflammation [4,5]. These T cells traverse the body through blood vessels of the circulatory system and are recruited by different factors. Pro-inflammatory cytokines, such as tumor necrosis factor alpha (TNFα) or interferon gamma (IFN-γ), induce adhesive properties of the endothelium, for example by means of upregulation of ICAM-1 [6]. In addition, directional migration is induced through gradients of chemokines such as CXCL12 [7]. Upon adhesion and encountering chemotactic cues, T cells can migrate across the vessel wall into underlying tissues through a complex multi-step process termed transendothelial migration (TEM) [8,9]. Extravasation from the endothelial vessel allows the T cells to elicit an immune response and could result in disease when its occurrence is either insufficient or excessive.

The use of mouse models enabled major breakthroughs in the field of immunology [10] and allows researchers to study immune cell dynamics in vivo [11,12,13,14,15]. Despite this, having vastly different immune systems raised the question as to what extent these models are translatable to humans [16]. Furthermore, they are expensive, labour intensive and tedious to work with as well as suffer from ethical issues [17]. Strong efforts are being made by the European Union to reduce, refine and replace animal models in science, called Directive 2010/63/EU [18]. In contrast, conventional in vitro cell culture does not recapitulate complex human biology [19], due to the lack of the vasculature, extracellular matrix (ECM) and neighbouring cells, and although the use of transwell systems has provided insights in the migratory behavior of various subsets of immune cells [20,21,22,23], migration in such systems is gravity-driven, while imaging and subsequent analysis can be tedious and thus of low-throughput [24].

Organ-on-chip technology provides the possibility to perform cellular assays with unprecedented control and flexibility [25]. Recently, several microfluidic models that allow the studying of immune cells have been described. Such advanced immune-competent in vitro models enable the recapitulation of varying immunological processes, e.g., monocyte adhesion [26], immune cell extravasation [27], neutrophilic infiltration [28], T cell- and NK cell-mediated anti-tumor responses [29,30,31] and the neutrophilic response to viral infection of human airways [32]. Although these models manage to recapitulate human biology better, thereby bridging the gap between in vivo models and conventional cell cultures, most are of low throughput, consist of PDMS and contain artificial membranes which makes them tedious to work with. 

The OrganoPlate, used in the abovementioned studies investigating monocyte-to-endothelium adhesion [26] and neutrophilic infiltration [28], is a platform which contains 40 microfluidic chips underneath a 384-well microtiter plate. Each chip can be prepared with a free-standing extracellular matrix gel, allowing a stratified culture setup devoid of artificial membranes [33]. Using this platform, we set out to develop an in vitro assay that can be used to study T cell dynamics in health and disease. We grow a blood vessel structure under perfused conditions [34] and apically add human primary T cells to the lumen thereof. Upon addition, T cell adhesion to the vessel wall and subsequent extravasation and migration into the adjacent ECM in dependence of various factors was studied, including activation state, presence of a chemotactic gradient and addition of inflammatory cytokines. We show that, although dynamics differ depending on their activation state, a combination of TNFα pretreatment and the presence of a CXCL12 gradient is a particularly powerful mix to induce TEM of both unstimulated and stimulated T cells. Furthermore, we studied the migration of T cells in conjunction with melanoma cells as a proof-of-concept to mimic immuno-oncological processes. In conclusion, we developed a comprehensive T cell extravasation assay comprising a perfused blood vessel, 3D ECM migration and a co-culture with tumor cells. We believe the assay will allow for gaining insight in T cell migratory behavior and can spur the development of novel therapies in the fields of immuno-oncology and auto-immunity. 

## 2. Results

### 2.1. Development and Characterization of 3D Endothelial Vessels Perfused with T Cells

**Figure 1:**
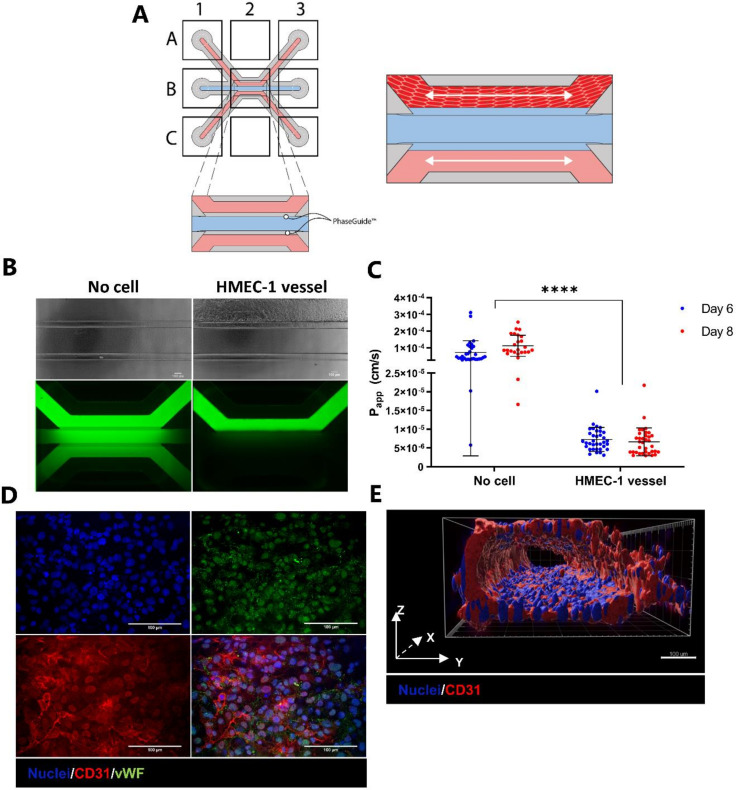
Endothelium on-a-chip. (**A**) A microfluidic chip, made up of a 3 by 3 grid, consists of three channels that come together in the center well (B2). The extracellular matrix (ECM, blue) is retained to the middle lane due to the PhaseGuide’s pressure barrier function. Endothelial cells can be seeded against the polymerized ECM after which a tubular structure is formed upon perfusion. (**B**) Perfusion of microfluidic chips with 10 kDa-sized FITC-dextran. The presence of a HMEC-1 vessel retains the fluorescent dextran to the top channel, demonstrating high barrier function (right). FITC-dextran has diffused throughout the chip when no vessel is present (left). (**C**) Barrier function was assessed at day 6 and day 8 after seeding by perfusing the vessels with medium containing fluorescently labeled dextran. Leakage of the fluorescent dye from the lumen of the vessel into the adjacent channel was measured using time-lapse imaging, and corresponding apparent permeability (P_app_) values (cm/s) were calculated. Shown are mean ± SD, and data points represent individual chips (*N =* 13, *n* = 2–4). Data were log transformed and analyzed using mixed effects analyses. Statistical analyses indicated significant differences in P_app_ values between chips containing HMEC-1 vessels and empty chips (**** *p* < 0.0001) and no significant difference between day 6 and day 8. (**D**) Immunofluorescent staining of endothelial vessels. Shown are maximum intensity projections; CD31 (red), Von Willebrand factor (green). Nuclei were counterstained using Hoechst 33342 (blue). (**E**) Three-dimensional reconstruction of CD31 (red) and nuclei (blue) showing a perfusable, tubular structure. Scale bar = 100 µm.

An endothelial vessel that is stable over the course of the experiment is a crucial prerequisite for an effective T cell extravasation assay. Endothelial vessels were grown using HMEC-1 cells in the OrganoPlate 3-lane (Figure 1A and Supplementary Figure S1A). To assess the barrier function of the vessel structures, HMEC-1 vessels were perfused with a medium containing 10 kDa-sized FITC-dextran. In contrast to no-cell controls, chips containing vessels retained fluorescent dye in the top lane (Figure 1B). Good barrier function was observed at day 6 after seeding HMEC-1 cells, as demonstrated by low apparent permeability (P_app_) values (Figure 1C). Barrier function remained unchanged in the T cell medium from day 6 to 8, providing a 48 h window for the co-culture with T cells (Figure 1C). Immunofluorescent staining revealed the expression of platelet endothelial cell adhesion molecule-1 (PECAM-1; CD31) and the Von Willebrand factor (vWF) (Figure 1D), both well-known markers for endothelial cells and, in the case of PECAM-1, a regulator of leukocyte trafficking [35]. Three-dimensional reconstruction verified the presence of a perfusable tubular structure with a clear luminal side (Figure 1E).

**Figure 2:**
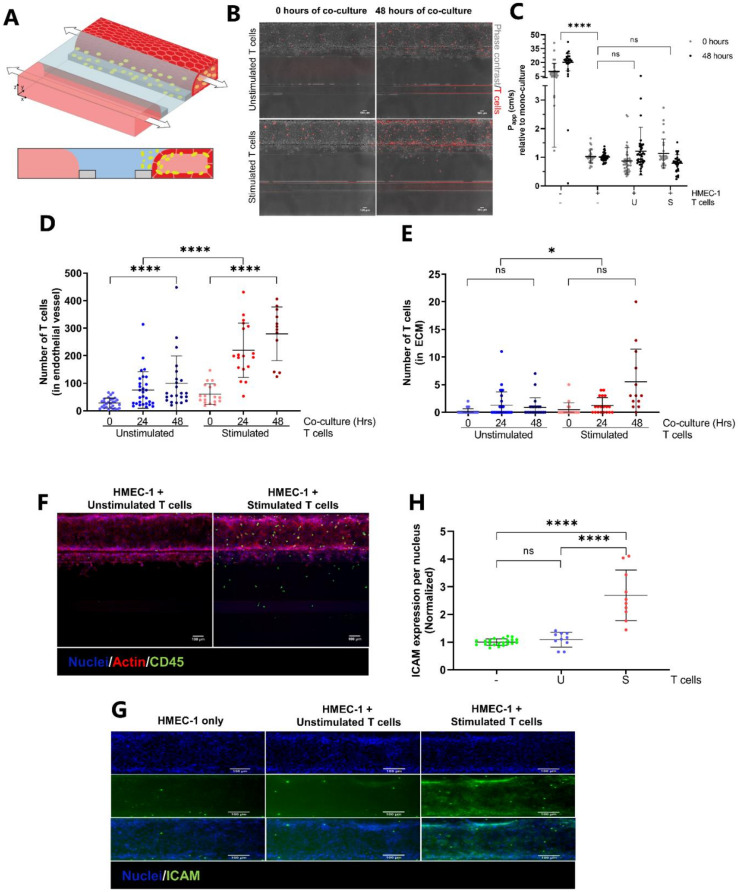
Characterization of a 3D co-culture comprising endothelial cells and T cells. (**A**) T cells (in yellow), unstimulated or stimulated with (CD3/CD28) beads, are added apically to the lumen of the HMEC-1 vessel. Upon perfusion, the T cells are able to undergo all steps of transendothelial migration (TEM); rolling, firm adhesion, crawling and migration across the endothelial vessel wall into the adjacent ECM. (**B**) Composite max intensity projections of live cultures consisting of HMEC-1 endothelial cells perfused with either unstimulated or stimulated fluorescently labelled T cells at the start and the end of 48 h co-culture. T cells (red, CellTracker Orange CMRA) reside inside the vessel and are able to extravasate into the adjacent ECM over time. (**C**) Barrier integrity of HMEC-1 vessels is not affected by co-culture with either unstimulated or stimulated T cells. Apparent permeability (Papp) values are normalized against Papp values of mono-cultured HMEC-1 vessels. Shown are mean ± SD, and data points represent individual chips (*N =* 8–13, *n =* 2–5). Statistical analysis was performed on non-normalized data. Data were log transformed and analyzed using mixed-effects models. Statistical analyses indicated significant differences between chips containing HMEC-1 vessels and empty chips (**** *p* < 0.0001; ns = not significant) and no significant differences between mono-cultured or co-cultured vessels. (**D**) Quantification of T cell numbers in the endothelial vessel after 0, 24 and 48 h of co-culture. Shown are mean ± SD, and data points represent individual chips (*N =* 4–7, *n =* 3–8). Data were log transformed and analyzed using Ordinary One-way ANOVA tests and mixed-effects models, showing a significant increase in number of T cells over time for both T cell populations (**** *p* < 0.0001) as well as a difference between unstimulated and stimulated T cells (**** *p* < 0.0001). (**E**) Quantification of T cell numbers in the ECM compartment after 0, 24 and 48 h of co-culture. Shown are mean ± SD, and data points represent individual chips (*N =* 4–7, *n =* 3–8). Data were log transformed and analyzed using Kruskall–Wallis tests and mixed-effects models, showing a significant difference between unstimulated and stimulated T cells (* *p* = 0.0206; ns = not significant). (**F**) Maximum intensity projections of co-cultures stained for CD31 (red) and CD45 (green). Nuclei are counterstained using Hoechst 33342. Scale bar = 100 µm. (**G**) Immunofluorescent staining of HMEC-1 vessels, either mono- or co-cultured, showing expression of ICAM-1. Shown are maximum intensity projections for nuclei (blue) and sum intensity projections for ICAM-1 (green). (**H**) Expression of Intercellular Adhesion Molecule 1 (ICAM-1) per HMEC-1 nucleus of mono-cultured vessels and vessels co-cultured with either unstimulated or stimulated T cells. Shown are mean ± SD normalized against mono-cultured HMEC-1 vessels. Data points represent individual chips (*N =* 4, *n =* 2–6). Data were log transformed and analyzed using Brown-Forsythe and Welch ANOVA tests showing a significant difference between the mono-culture and co-culture using stimulated T cells as well as between the two co-cultures (**** *p* <0.0001; ns = not significant).

HMEC-1 vessels were perfused with either unstimulated or stimulated T cells and co-cultured for 48 h (Figure 2A). Before addition, both T cell populations showed a similar % of CD3+, CD4+ and CD8+ cells, although functional differences in IFNγ production were observed (Table 1 and Table 2 and Supplementary Figure S2). Stimulated and unstimulated T cells had no negative effect on the HMEC-1 barrier function, indicated by the comparable P_app_ values relative to mono-cultured vessels (Figure 2C). T cell behavior was followed in real time using CellTracker dye. Quantification of T cells in the vessel compartment showed a significantly increased number of T cells over time for both unstimulated and stimulated T cells. However, the number of stimulated T cells attached to the HMEC-1 vessel was significantly higher over time compared to unstimulated T cells (Figure 2D). 

Next, we determined whether T cells extravasated from the endothelial vessels. Quantification of fluorescent T cells in the ECM compartment showed that T cells of both populations extravasate into the ECM although in low numbers (Figure 2E). However, stimulated T cells migrated more frequently (* *p* = 0.0206). Immunofluorescent staining for CD45 confirmed the presence of T cells in the vessel and in the adjacent ECM (Figure 2F and Supplementary Figure S3). Unstimulated T cell numbers in the endothelial vessel visualized by CellTracker dye versus CD45 staining differ greatly (Figure 2D vs. Figure 2F) due to the staining procedure, involving repeated washing steps, which may affect loosely attached unstimulated T cells. Staining for ICAM-1 showed unchanged ICAM-1 expression per nucleus when HMEC-1 vessels were co-cultured with unstimulated T cells compared to mono-cultured vessels (Figure 2G,H). In contrast, perfusing vessels with stimulated T cells resulted in a significant 2.7-fold increase of ICAM-1 expression.

### 2.2. CXCL12 Stimulates Transendothelial Migration of T Cells into Collagen Hydrogel 

**Figure 3:**
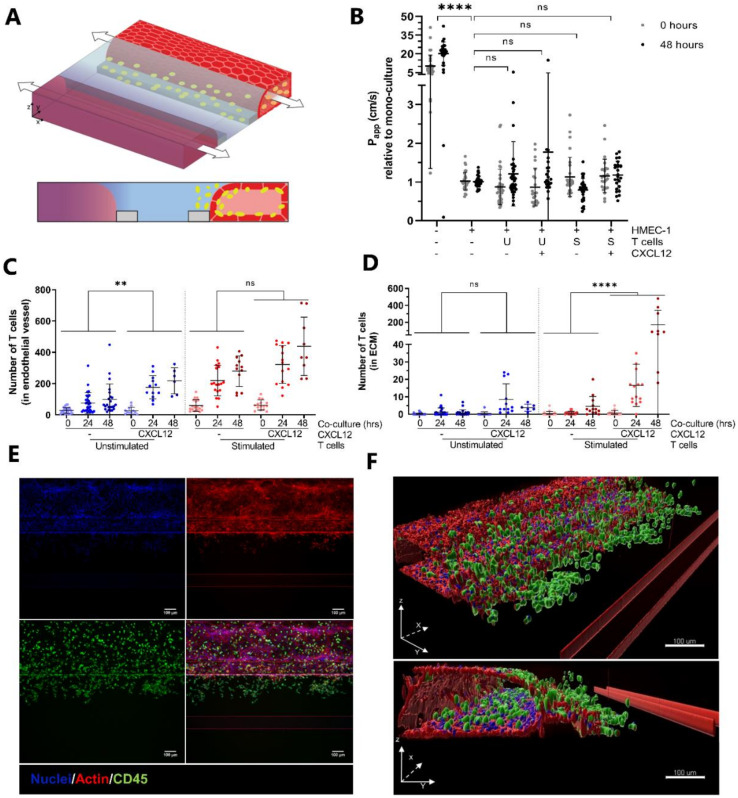
Addition of the chemokine CXCL12 increases T cell migration. (**A**) A chemotactic gradient is formed upon perfusion of the bottom channel with medium containing CXCL12 (purple hue). (**B**) Barrier integrity of HMEC-1 vessels is not affected by co-culture with either unstimulated or stimulated T cells in the presence or absence of CXC12. Apparent permeability (Papp) values are normalized against Papp values of mono-cultured HMEC-1 vessels. Shown are mean ± SD, and data points represent individual chips (*N =* 7–13, *n =* 2–5). Statistical analysis was performed on non-normalized data. Data were log transformed and analyzed using mixed-effects models. Statistical analyses indicated significant differences between chips containing HMEC-1 vessels and empty chips (**** *p* < 0.0001), and no significant (ns) differences between mono-cultured and co-cultured vessels with or without CXCL12. (**C**) Quantification of T cell numbers in the endothelial vessel after 0, 24 and 48 *h* of co-culture. Shown are mean ± SD, and data points represent individual chips (*N =* 2–7, *n =* 3–8). Data were log transformed and analyzed using mixed-effects models showing a significant effect of CXCL12 on the number of unstimulated T cells (** *p* = 0.0026; ns = not significant) residing in the vessel. (**D**) Quantification of T cell numbers in the ECM compartment after 0, 24 and 48 *h* of co-culture. Shown are mean ± SD, and data points represent individual chips (*N =* 2–7, *n =* 3–8). Data were log transformed and analyzed using mixed-effects models showing a significant effect of CXCL12 on the migration of stimulated T cells (**** *p* < 0.0001; ns = not significant). (**E**) Maximum intensity projections of HMEC-1 vessels co-cultured with stimulated T cells in the presence of CXCL12 stained for actin (red) and CD45 (green). Nuclei were counterstained using Hoechst 33342 (blue). (**F**) Three-dimensional reconstruction of actin (red), CD45 (green) and nuclei (blue) showing the attachment of T cells to and migration across the endothelial vessel wall. Scale bar = 100 µm.

Based on the results described above, it seemed that the migration of T cells, either unstimulated or stimulated, occurred only in low numbers under basal conditions. To investigate whether T cells in our co-culture model could be triggered to migrate by the addition of chemo-attractants, the chemokine CXCL12 was added to the bottom perfusion channels at the start of the co-culture (Figure 3A, purple hue). CXCL12 is a ligand for CXCR4, expressed by leukocytes, and extensively used in immunology research [7]. The addition of CXCL12 did not affect vascular permeability, indicated by the comparable relative P_app_ values (Figure 3B). The quantification of T cell numbers showed that CXCL12 significantly increased the number of unstimulated T cells residing in the vessel at 24- and 48-h timepoints (Figure 3C). Interestingly, the number of stimulated T cells migrating into the ECM increased greatly in response to the addition of CXCL12, while the migration of unstimulated T cells was hardly affected (Figure 3D). Although the addition of CXCL12 undeniably affects the migration of stimulated T cells, the extent to which it does varies greatly between donors as shown in Supplementary Figure S4. Immunofluorescent staining for CD45 verified the presence of an increased number of stimulated T cells in the ECM compartment in response to CXCL12 (Figure 3E,F).

### 2.3. Transendothelial Migration of T Cells Is Increased by Addition of CXCL12 under Inflammatory Conditions

**Figure 4:**
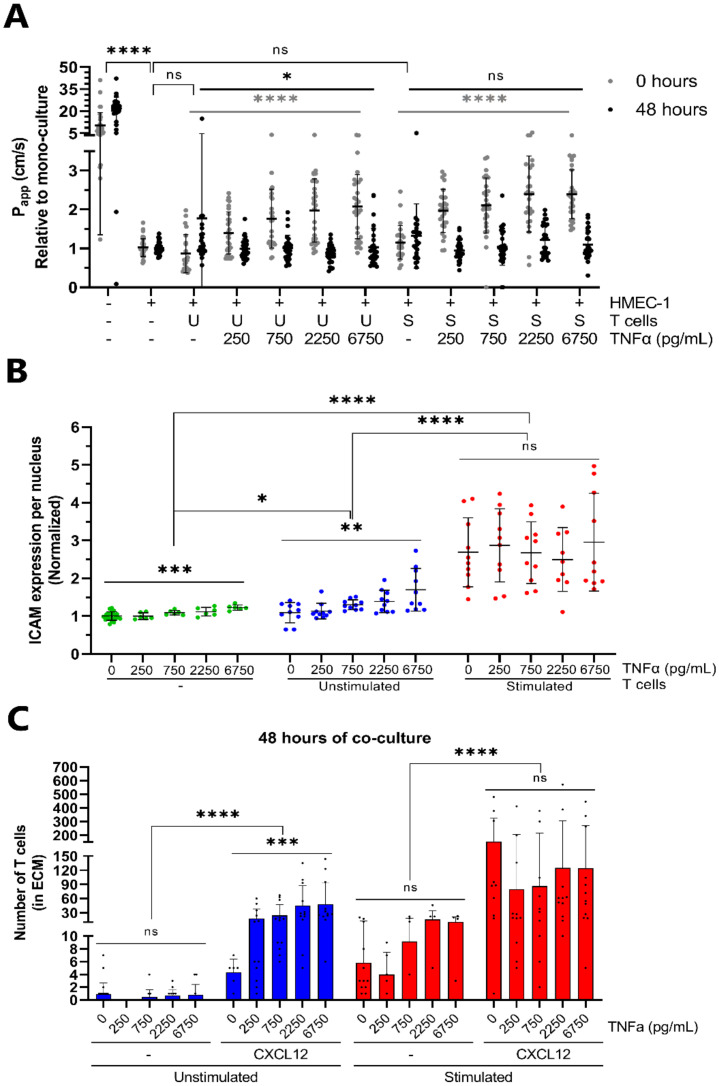
Inflammation potentiates migration of unstimulated T cells in response to CXCL12. (**A**) Barrier integrity of HMEC-1 vessels is affected by TNFα pretreatment. Apparent permeability (Papp) values are normalized against Papp values of mono-cultured HMEC-1 vessels. Shown are mean ± SD, and data points represent individual chips (*N =* 6–13, *n =* 2–7). Statistical analysis was performed on non-normalized data. Data were log transformed and analyzed using mixed-effects models indicating significant differences between chips containing HMEC-1 vessels and empty chips (**** *p* < 0.0001) and no significant difference between mono-cultured HMEC-1 vessels and vessels co-cultured with either unstimulated T cells (U) or stimulated T cells (S). Effect of inflammation on barrier function was assessed by analyzing transformed data using Kruskall–Wallis tests showing a significant effect of TNFα pretreatment at 0 h (bold grey, **** *p* < 0.0001) and no (S) or a reduced effect (U) at 48 h after co-culture with respectively stimulated and unstimulated T cells (bold black, ns = *p* > 0.05 and * *p* = 0.0360 respectively). (**B**) Expression of Intercellular Adhesion Molecule 1 (ICAM-1) per HMEC-1 nucleus of mono-cultured vessels and vessels co-cultured with either unstimulated or stimulated T cells in response to TNFα pretreatment. Shown are mean ± SD, and data points represent individual chips (*N =* 3, *n =* 2–6). Data were normalized against mono-cultured, non-treated HMEC-1 vessels, and dose-responses were analyzed after log transformation using Brown–Forsythe and Welch ANOVA tests, showing a significant concentration-dependent effect of TNFα pretreatment on ICAM-1 expression for mono-cultured vessels (*** *p* = 0.0001) and vessels co-cultured with unstimulated T cells (** *p* = 0.0019). Differences between mono-culture and co-cultures were analyzed using Two-way ANOVA tests after log transformation, showing a significantly different response to TNFα pretreatment between the mono-cultured vessels and vessels co-cultured with unstimulated T cells (* *p* = 0.0120) and stimulated T cells (**** *p* < 0.0001) as well as between both co-cultures (**** *p* < 0.0001). (**C**) Quantification of T cell numbers in the ECM compartment after 48 h of co-culture in response to TNFα pretreatment in the presence or absence of CXCL12. Shown are mean ± SD, and data points represent individual chips (*N =* 2–7, *n =* 3–5). Data were analyzed using One-way and Two-way ANOVA tests after log transformation, showing a significant effect of CXCL12 on the migration of T cells after TNFα pretreatment for both unstimulated (blue) and stimulated (red) T cells (**** *p* < 0.0001) as well as a TNFα effect on migration of unstimulated T cells in the presence of CXCL12 (*** *p* = 0.0008). ns = not significant.

Inflammation occurs in vivo upon tissue damage, which can be due to physical, chemical or biological insults, and results in the alteration of the vasculature and recruitment of leukocytes [36]. Therefore, we investigated whether mimicking inflammatory conditions in our model would affect the behavior of T cells in the co-culture. HMEC-1 vessels were incubated with a concentration range (0–6750 pg/mL) of TNFα prior to co-culture with T cells. The barrier function of vessels decreased after TNFα pretreatment, indicated by increasing relative P_app_ values with TNFα concentration, but recovered over the 48-h co-culture period (Figure 4A). ICAM-1 staining showed enhanced expression of ICAM-1 per HMEC-1 nucleus with increasing TNFα concentration (Figure 4B and Supplementary Figure S5), with a minor increase in the case of mono-cultured vessels (green, 1.0–1.23, *** *p* = 0.0001) and a more prominent increase in the case of vessels co-cultured with unstimulated T cells (blue, 1.09–1.70, ** *p* = 0.0019). Although there is a significant difference between mono-cultured vessels and both co-cultures (unstimulated: * *p* = 0.0120, stimulated: **** *p* < 0.0001), ICAM-1 expression of vessels co-cultured with stimulated T cells was not affected by TNFα pretreatment but was constitutively elevated (red, 2.50–2.96, *p* = 0.9166). Interestingly, TNFα pretreatment affected ICAM-1 expression of HMEC-1 vessels differently depending on whether vessels were perfused with unstimulated or stimulated T cells (**** *p* < 0.0001). 

Pretreatment of endothelial vessels with TNFα did not result in clear differences in the number of unstimulated or stimulated T cells in the HMEC-1 vessels (Supplementary Figure S6A,B). Moreover, the quantification of T cell numbers in the ECM compartment showed no TNFα-dependent increase in the extravasation of unstimulated and stimulated T cells after 24 (Supplementary Figure S6C) and 48 h (Figure 4C) of co-culture. However, TNFα pretreatment in combination with the addition of CXCL12 increased the migration of unstimulated T cells in what seemed to be a concentration-dependent effect (Figure 4C, blue bars, *** *p* = 0.0008). Between 24 and 48 h of co-culture, the migration of stimulated T cells increased greatly in response to CXCL12, while the number of migrating unstimulated T cells hardly increased during this period (Supplementary Figures S4C and S6C). However, this increase in migrating stimulated T cells mediated by CXCL12 was not affected by TNFα pretreatment (*p* = 0.4008). Regression analysis verified different responses to TNFα pretreatment depending on stimulation, the presence of CXCL12 and the duration of co-culture (Supplementary Figure S7).

### 2.4. Proof-of-Concept; Transendothelial T Cell Migration towards A375 Melanoma Cells

**Figure 5:**
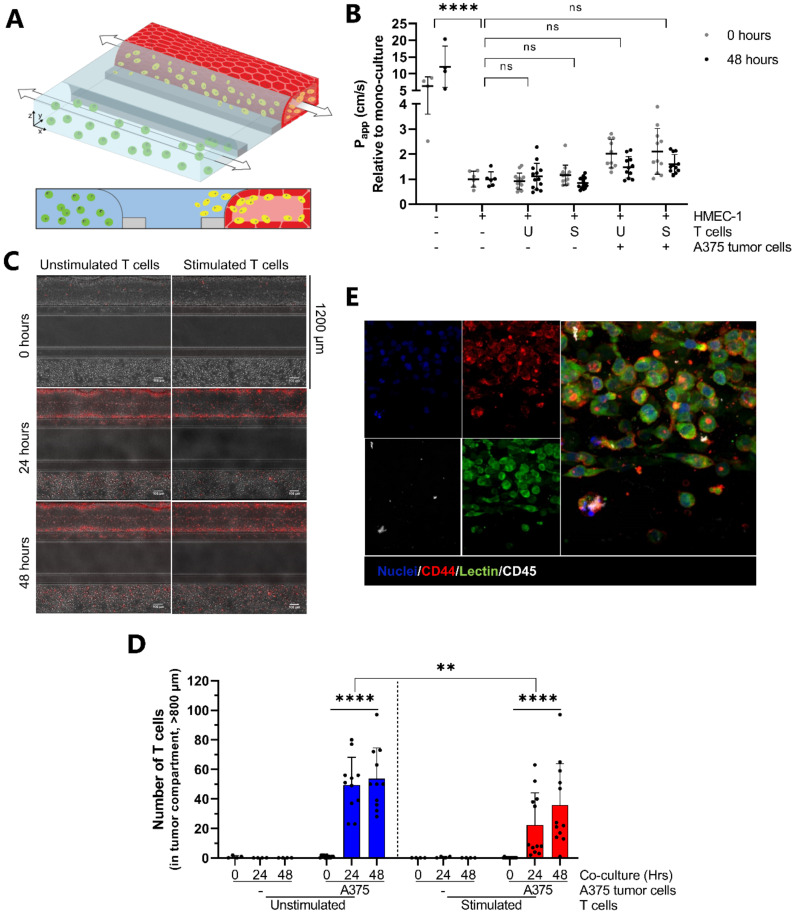
The migration of T cells is affected by the presence of A375 dermal melanoma cells. (**A**) A375 tumor cells (green) embedded in a second ECM gel are patterned in the bottom channel prior to the addition of T cells (yellow) to the apical side of the HMEC-1 vessel. (**B**) The presence of A375 tumor cells slightly affects barrier integrity of the HMEC-1 vessel. Apparent permeability (Papp) values are normalized against Papp values of chips containing mono-cultured HMEC-1 vessels and an empty ECM gel in the bottom channel. Shown are mean ± SD, and data points represent individual chips (*N =* 2, *n =* 2–6). Statistical analysis was performed on non-normalized data. Data were log transformed and analyzed using mixed-effects models. Statistical analyses indicated significant differences between chips containing HMEC-1 vessels and empty chips (**** *p* < 0.0001) and no significant differences (ns) between mono-cultured or co-cultured vessels. (**C**) Composite max intensity projections of live cultures consisting of HMEC-1 endothelial cells perfused with either unstimulated or stimulated fluorescently labelled T cells (red, CellTracker Orange CMRA) after 0, 24 and 48 h of co-culture. The bottom channel of the microfluidic chip contains ECM-embedded A375 dermal melanoma cells. Scale bar = 100 µm. (**D**) Unstimulated and stimulated T cells migrate towards A375 dermal melanoma cells. The positions of individual T cells throughout microfluidic chips were determined along the width of the chips, which measures 1200 µm in total. T cells that migrated >800 µm were considered to reside in the tumor compartment. Shown are mean ± SD, and data points represent individual chips (*N =* 2, *n =* 2–6). Data were analyzed using Two-way ANOVA tests and Kruskal–Wallis tests after log transformation, showing a significant effect of A375 melanoma cells on the migration of both T cell populations (**** *p* < 0.0001) as well as a significant difference between unstimulated and stimulated T cells (** *p* < 0.0012) (**E**) Immunofluorescent staining of the bottom compartment of a microfluidic chip containing A375 dermal melanoma cells showing co-localization of a T cell with a fragmented A375 dermal melanoma cell. 20× magnification, shown are maximum intensity projections; CD44 (red), Lectin (green) and CD45 (white). Nuclei were counterstained using Hoechst 33342 (blue).

Currently, the field of immuno-oncology is of high interest due the success of certain immunotherapies [37]. To investigate whether our co-culture model could be used for immuno-oncology studies, a proof-of-concept study was performed in which we incorporated tumor cells into our assay setup. A second ECM gel, either empty or containing A375 melanoma cells, was added to the bottom channel 16 h prior to perfusion of the HMEC-1 vessel with T cells (Figure 5A). Although not statistically significant, HMEC-1 vessels in chips containing malignant melanoma A375 cells showed slightly increased relative P_app_ values, indicating that the tumor cells affect vascular permeability (Figure 5B). CellTracker dye was used to determine the positions of T cells within the microfluidic chips along the Y-axis. Both unstimulated and stimulated T cells migrated towards the tumor cells within 24 h of co-culture, indicated by the co-localization of tumor cells and a fluorescent signal from the T cells (Figure 5C). Quantification showed that, although the migration of both T cell populations was increased, higher numbers of unstimulated T cells migrated towards the A375 cells (Figure 5D, blue vs. red). However, at 48 h, a second wave of migrating stimulated T cells was observed in the ECM compartment (400 µM–800 µm) (Supplementary Figure S8, dark blue vs. dark red). CD45-positive staining was observed in the tumor compartment and seen to co-localize with a fragmented cell nucleus (Figure 5E). 

## 3. Discussion

In this study, we describe a novel 3D T cell extravasation and migration assay using a high-throughput microfluidic platform. The assay captures the attachment of T cells to an endothelial vessel under flow and extravasation from this vessel wall into the underlying tissue. The HMEC-1 cell line was used to grow endothelial vessels against an ECM which, upon exhibiting high barrier function, was perfused with fluorescently labelled T cells. The co-culture was stable for at least 48 h during which the migration of T cells across the endothelium was observed. The migration of T cells could be induced by adding the chemotactic trigger CXCL12 or a co-culture with the melanoma cell line A375 into the bottom perfusion lane. The fact that T cell migration is unimpeded by artificial membranes, the presence of a 3D ECM-like scaffold as well as the multi-cellular nature of this model are crucial requirements for mimicking in vivo-like conditions [19].

In migration studies using transwell systems, gravity and gradient instability causes a narrowed time window and makes long-term assessment of the effects of chemotactic triggers difficult [38,39,40]. Recently, there have been multiple reports of novel 3D in vitro immune-competent models, among which some are used to study T cell dynamics [29,30]. The model described in this study, however, has several benefits. Most models suffer from complex procedures for cell seeding and maintenance, often contain polydimethylsiloxane (PDMS) [41,42,43] as well as artificial membranes and do not allow easy imaging. Furthermore, the incorporation of vasculature and perfusion flow in these models often is not straightforward which is imperative when modelling TEM [44]. The OrganoPlate utilizes optical quality glass and polymers that are biocompatible and low compound-absorbing and is devoid of artificial membranes. Furthermore, perfusion flow can be achieved without the need of pumps. Although being bidirectional and not unidirectional, the presence of the perfusion flow makes the model more relevant than static alternatives [45]. Being easy-to-use and compatible with standard lab equipment while containing 40 individual microfluidic chips, the platform is highly suitable for automated, high-throughput research. The numbers of added T cells that attach to the endothelium and migrate into the ECM might seem to be lower compared to other T cells migration models. This can be explained by the fact that each channel of the microfluidic chip is connected to two wells of the 384 well plate that act as medium reservoirs. The 20,000 T cells are added in total of 100 µL of medium, but only 1–2 µL of this volume actually enters the microfluidic channel, which means that only 200–400 T cells should end up in the endothelial vessel. So, although the number of T cells might seem low, it is in the range of what is expected based on the numbers that are added and considering the geometry of the microfluidic chip. 

The role of chemokines as regulators and drivers of T cell trafficking has been appreciated for a long time [46]. The addition of the chemokine CXCL12 and the formation of a chemotactic gradient in our co-culture model resulted in a significant increase in migrating stimulated T cells. Although there was a minor increase, the effect of CXCL12 on the migratory behavior of unstimulated T cells was not significant. This can be explained by the observation that perfusion with stimulated T cells increased ICAM-1 expression, an important regulator of TEM [47] of the endothelial vessel, while perfusion with unstimulated T cells did not. Elevated ICAM-1 expression in co-culture with stimulated T cells is likely caused by an increased production of pro-inflammatory cytokine IFNγ by stimulated T cells [48]. Without a chemotactic trigger, only occasional T cell migration across the endothelium was observed over 48 h. This is similar to the in vivo situation, where T cells mainly stay in the blood vessel in the absence of a cue to extravasate [8]. 

Inflammation results in the increased homing of T cells towards the site of insult in vivo [49]. Mono-cultured HMEC-1 vessels treated with TNFα resulted in an inflammatory phenotype, as became apparent from the upregulation of ICAM-1 expression and the increase in vascular permeability [50,51]. TNFα pretreatment of HMEC-1 vessels prior to co-culture with unstimulated T cells showed a concentration-dependent increase in ICAM-1 expression to a significantly higher extent compared to the concentration-dependent increase observed for mono-cultured HMEC-1 vessels. In contrast, co-culturing vessels with stimulated T cells resulted in a higher expression of ICAM-1 which was not further increased by TNFα pretreatment prior to co-culture, suggesting that perfusion with stimulated T cells already induced an inflammatory environment. The number of migrating unstimulated T cells did not increase in response to TNFα pretreatment alone but resulted in a concentration-dependent increase when combined with the CXCL12 addition. We hypothesize that the migration of T cells towards a CXCL12 gradient occurs under inflammatory circumstances, and treatment with TNFα therefore possibly potentiates unstimulated T cell migration by compensating for the difference in secretion of pro-inflammatory cytokines between the two different T cell populations. Secretion of pro-inflammatory cytokines by stimulated T cells probably induces an inflammatory phenotype of the HMEC-1 vessel, making the effect of TNFα pretreatment negligible, resulting in the migration of similar numbers of stimulated T cells in response to CXCL12 regardless of the TNFα concentration. This hypothesis is supported by the observation that both populations of T cells were seen to migrate towards A375 tumor cells, as the tumor microenvironment is characterized by the secretion of a plethora of pro-inflammatory cytokines and chemokines [52,53]. 

Although not statistically significant, the presence of the malignant melanoma A375 cell line in our co-culture model resulted in increased vascular permeability, a hallmark of inflammation and melanoma vasculature [54]. CD45-positive cells were detected in the tumor compartment, although in lower numbers than when compared to quantification by means of CellTracker dye. The presence of a second hydrogel in the bottom channel, which hampers immunofluorescent staining due to a lack of flow resulting in reduced penetration of certain antibodies, is likely responsible for this difference. In a few cases, co-localization of CD45-positive staining with fragmented tumor cell nuclei was observed. Although difficult to determine the cause of fragmentation, it raises the question of whether the T cells in this model are able to kill the A375 tumor cells. Other in vitro models report the occurrence of tumor cell killing within 24 to 72 h of co-culture [29,30]. The 48 h co-culture is possibly not sufficient to induce T cell-mediated tumor cell killing in our setting, considering the migration distance and the T cells not being primed towards the A375 melanoma cells.

Thus, extending the co-culture duration in the future could possibly allow the assessment of T cell-mediated tumor cell killing in our assay. Furthermore, we expect that the use of donor matched cells will have a major impact on the quality of the assay. A donor matched setup can be established by using primary T cells and blood outgrowth endothelial cells (BOECs) [55,56], both isolated from PBMCs as well as patient-derived tumor material. Such a donor matched co-culture could be used to predict individual responsiveness to immunotherapeutics and determine optimal treatment strategies. This provides a unique opportunity since the immune system is known to be complex and immensely diverse, showing huge variation within and between individuals [57]. Indeed, looking at the contribution of different donors to the acquired data in our study, T cells isolated from certain donors tend to show higher numbers of migrating T cells which underlines this donor-to-donor variation (Supplementary Figure S4). 

Although the assay could well be applicable to more long-term experiments, the labelling agent that was used in this study is not. We observed differences in labelling intensity after a 48 h co-culture and therefore were forced to exclude a few chips to assure the reliable quantification of T cell numbers, resulting in fewer data points for certain conditions over time. Despite this, the CellTracker Orange CMRA label was considered sufficient to describe and characterize the 48 h co-culture in our study. In future work, we aim for the use of a more stable fluorescent label over time in order to prolong the assay window. 

In conclusion, we developed an assay for the routine study of transendothelial T cell migration in 3D. The assay comprised a perfused vascular component that allowed a stable arrest of T cells, followed by extravasation and migration into a 3D extracellular matrix. Similar to in vivo, the occurrence of these events in the assay are modulated by inflammation and the presence of chemotactic gradients. Co-culture with tumor cells enabled studying T cell invasion into tumor microenvironments and their interaction. The assay is based on a microfluidic platform, allowing for the execution of 40 assays in parallel that are fully imaging and automation compatible. We envision the assay for routine use to understand immunological processes and disease better, ultimately leading to novel therapies.

## 4. Materials and Methods

### 4.1. Cell Culture

The 2D cell culture of HMEC-1 endothelial cells and A375 melanoma cells and isolation of primary CD3+ T cells are described in Supplementary Materials.

### 4.2. T Cell Stimulation and Labelling

CD3+ T cells were cultured in 6-well plates (Costar, 734-1599) in AIM-V medium (Invitrogen, 12055091, Waltham, MA, USA) with or without CD3/CD28 Human T-activator Dynabeads (Gibco, 11161D, Waltham, MA, USA) at a bead-to-T-cell ratio of 1:10 for 48 h, resulting in stimulated and unstimulated T cells, respectively. In experiments with A375 tumor cells, T cells were cultured in 6-well plates, coated with 1 µg/mL anti-CD3 (Biolegend, 317326, San Diego, CA, USA), with 0.25 µg/mL anti-CD28 (Biolegend, 302914) to achieve stimulation. 

T cells were labelled with CellTracker Orange CMRA (Invitrogen, C34551). A total of 5 mM CellTracker were prepared in 18 µL dimethylsulfoxide (DMSO, Sigma, D8418, St. Louis, MO, USA), followed by a working solution of 2.5 µM prepared in phosphate buffered saline (PBS, Thermo Fischer Scientific, 70013016, Waltham, MA, USA). T cells were harvested and pelleted (300× *g*, 5 min) before being resuspended in 2 mL working solution, vortexed and incubated in the dark in a water bath (37 °C) for 30 min. Staining was terminated by the addition of a 10 mL AIM-V medium. T cells were pelleted (300× *g*, 5 min) and resuspended in AIM-V medium.

### 4.3. Microfluidic Co-Culture

The OrganoPlate 3-lane (MIMETAS, 4003-400B, Leiden, The Netherlands), containing 40 chips with, respectively, 9 mm and 12.2 mm long gel and perfusion channels (400 µm × 220 µm (w × h)), was used (Figure 1A and Supplementary Figure S1A). The loading of extracellular matrix (ECM) gel, cell seeding and the principle behind PhaseGuide functioning were previously described [33]. HMEC-1 cells at a density of 1 × 10^7^ cells/mL were used. In the case of migration studies under inflammatory conditions, the culture medium was replaced with AIMV containing a concentration range (0–6750 pg/mL) of recombinant tumor necrosis factor α (TNFα, R&D Systems, 210-TA-020, Minneapolis, MN, USA) 16 h prior to co-culture. In the case of migration studies in the presence of a tumor cell compartment, 1 × 10^7^ cells/mL A375 tumor cells were resuspended in a 4 mg/mL collagen I gel, and 2 µL were seeded in the bottom perfusion channels of microfluidic chips 16 h prior to co-culture. 

To set up T-cell-endothelium co-cultures, the endothelial culture medium was aspirated at day 6, and 50 µL of T cell suspension containing 20,000 T cells were added to the top perfusion channel inlets followed by the addition of 50 µL AIM-V medium to the outlets. A total of 50 µL of either plain AIM-V medium or AIM-V medium containing 800 ng/mL CXCL12 (Peprotech, 300-28A, Rocky Hill, NJ, USA) were added to the inlet and outlet of the bottom perfusion channel, and the plate was incubated (37 °C, 5% CO_2_) on a rocking platform (8-min interval, 7° inclination) for 48 h. The co-culture was imaged at 0, 24 and 48 h of co-culture using the Molecular Devices ImageXpress Micro Confocal High-Content Imaging System (Molecular Devices, San Jose, CA, USA). 

### 4.4. Barrier Integrity Assessment

The barrier integrity of HMEC-1 vessels was assessed before the T cell addition and at the end of the co-culture using a fluorescent dye as previously described [58]. More information can be found in the supplementary materials.

### 4.5. Quantification of T Cell Dynamics

Images were acquired using the 60 µm spinning disc confocal mode, a 0.45NA dry-air 10× objective and dichroic/emission fluorescent filters for TRITC. Maximum projections of fluorescently labelled T cells were generated and used to determine positions (top to bottom) of T cells in the microfluidic chips using FIJI (version 2 build 1.52d). Images were corrected for photobleaching-induced artifacts, and a rolling ball background correction was applied to improve the signal-to-noise ratio [59]. An automatic thresholding routine was used to create binary masks of fluorescently labelled T cells within images, and a particle detection algorithm was applied resulting in individual T cells being outlined and labelled [60]. The list was used to extract X/Y positional information about individual T cells within microfluidic chips. T cells quantified at Y positions ≤ 450 µm were considered to reside in endothelial vessels, while T cells quantified at Y positions > 450 µm were considered migrated T cells. 

### 4.6. Statistical Analysis

Data were tested for normality using QQ-plots and normality tests and log-transformed if needed. Statistically significant differences between means of two or more groups were assessed using one-way ANOVA (Gaussian, homogeneity of variance), Brown-Forsythe and Welch ANOVA (Gaussian, heterogeneity of variance) or Kruskal–Wallis tests (non-Gaussian). In the case of two factors, two-way ANOVA tests were performed. Multiple comparisons were accounted for using Tukey’s or Dunnett’s tests. Statistical analyses were performed in GraphPad Prism v8.0 (GraphPad Software, San Diego, CA, USA). Differences were considered significant when *p* < 0.05. Independent experiments are denoted by *N*, while replicates per experiment are denoted by *n*.

## Figures and Tables

**Table 1:** Characterization of T cell populations with flow cytometry ^1^.

	Frequency of Parent	
Subset	Unstimulated T cells	Stimulated T cells
Single cells	97.75 ± 0.55%	88.95 ± 0.75%
CD14+, CD3+	4.55 ± 0.15%	6.17 ± 0.08%
CD14+, CD3-	1.69 ± 0.36%	2.51 ± 0.28%
CD14−, CD3+	91.20 ± 0.80%	86.15 ± 1.35%
CD8+, CD4+	0.11 ± 0.02%	1.08 ± 0.07%
CD8+, CD4-	20.10 ± 0.70%	19.80 ± 1.0%
CD8−, CD4+	78.05 ± 1.45%	77.60 ± 1.4%
CD8−, CD4-	1.77 ± 0.70%	1.50 ± 0.45%
CD14−, CD3	2.60 ± 0.27%	5.17 ± 1.02%

^1^ Classification of different subsets for unstimulated and (CD3/CD28) stimulated T cells based on flow cytometry data. Bold numbers highlight CD3+, CD8+ and CD4+ single positive subsets. *N =* 2.

**Table 2:** Comparison of pro-inflammatory cytokine secretion between T cell populations ^1^.

	Frequency of Parent	
Subset	Unstimulated T cells	Stimulated T cells
Single cells	98.45 ± 0.35%	94.65 ± 0.35%
Live cells	92.20 ± 0.20%	94.60 ± 0.10%
IFNγ+	0.19 ± 0.13%	38.45 ± 2.85%
IFNγ-	99.82 ± 0.12%	61.55 ± 2.85%

^1^ Secretion of interferon gamma (IFNγ) of unstimulated and (CD3/CD28) stimulated T cells was assessed using intracellular staining. Bold numbers highlight IFNγ+ subset. *N =* 2.

## Data Availability

Not applicable.

## Supplementary Materials

The following are available online at https://www.mdpi.com/article/10.3390/ijms22158234/s1.

## Abbreviations

ECM	Extracellular matrix
TNFα	Tumor necrosis factor alpha
IFNγ	Interferon gamma
ICAM-1	Intercellular adhesion molecule
TEM	Transendothelial migration
FITC	Fluorescein isothiocyanate
TRITC	Tetramethylrhodamine isothiocyanate
PECAM-1	Platelet endothelial cell adhesion molecule-1
vWF	Von Willebrand factor
PDMS	Polydimethylsiloxane
PBMC	Peripheral blood mononuclear cell
