# Supplementary material for: A Microfluidic 3D Endothelium-on-a-Chip Model to Study Transendothelial Migration of T Cells in Health and Disease
Source: Int J Mol Sci. 2021 Jul 30;22(15):8234. doi: 10.3390/ijms22158234 (PMC8347346; doi:10.3390/ijms22158234)
Supplement: Supplementary file 1 [file ijms-22-08234-s001.zip › ijms-1304768-supplementary.pdf]

International Journal of *Molecular Sciences*

MDPI

Supplementary Material & Methods

2D Cell Culture

HMEC-1 a human dermal microvascular endothelial cell line (ATCC, CRL-3243, Manassas, VA, USA) was cultured in T-75 flasks (Corning, 431464U, Corning, NY, USA) using MCDB131 medium (Thermo Fischer Scientific, 10372019, Waltham, MA, USA) supplemented with 10% Fetal Bovine Serum (ATCC, 30-2020), 10 ng/mL human Epidermal Growth Factor (hEGF, Sigma, E9644, St. Louis, MO, USA), 1 µg/mL hydrocortisone (Sigma, H0135), 10 mM L-Glutamine (Sigma, G7513) and 1% Penicillin-Streptomycin (Sigma, P4333), further referred to as endothelial culture medium. HMEC-1 cells were used at passage number 27. Before use, HMEC-1 cells were harvested using 0.25% trypsin (Gibco, 15090-046, Waltham, MA, USA), pelleted (100× *g*, 5 min) and resuspended in endothelial culture medium.

A375 a human dermal melanoma cell line (ATCC, CRL-1619) was cultured in T-75 flasks using RPMI medium (Thermo Fischer Scientific, 11879020, Waltham, MA, USA) supplemented with 10% Fetal Bovine Serum (Gibco, 16140-071), 2 mM L-Glutamine (Sigma, G7513) and 1 mM sodium pyruvate (Thermo Fischer Scientific, 11360070), further referred to as tumor cell medium. A375 cells were used until passage 12. Before use, A375 cells were harvested using 0.25% trypsin, pelleted (200× *g*, 5 min) and resuspended in tumor cell medium. All cell culture was performed in a humidified incubator at 37 °C and 5% CO2.

T Cell Isolation

T cells were isolated from human buffy coats of healthy donors obtained from the Dutch blood bank Sanquin. First, Peripheral Blood Mononuclear cells (PBMCs) were isolated from the buffy coats by a density gradient centrifugation method using 1.078 g/mL ficoll (GE Healthcare, 17-1440-02, Chicago, IL, USA) and Leucosep™ tubes (VWR, 720-1840, Radnor, PA, USA). PBMCs were stored in liquid nitrogen until further use. PBMCs were thawed and CD3+ T cells were isolated using the EasySep™ Human T cell Isolation Kit (Stemcell Technologies, 17951, Vancouver, Canada).

T cells used in co-culture experiments with A375 tumor cells were isolated from whole blood from healthy donors acquired at Merck's medical department (Merck Healthcare KGaA, Darmstadt, Germany). Whole blood was diluted with washing buffer (PBS + 2% FBS) and gently layered overall an equal volume of Biocoll (Biochrom, #L6115, Holliston, MA, USA) in a SepMate (StemCell Technologies, #85460) tube and centrifuged (1200 x g, 20 min) without brake resulting in the formation of four layers. The second layer containing the PBMCs was gently removed, washed with washing buffer and spun down (300× *g*, 8 min). Cells were washed once more and centrifuged (120 x g, 10 min) without brake to remove remaining platelets. Pelleted cells were resuspended in buffer and counted using ViCell counter. T cells were subsequently isolated using the standard Pan T cell isolation kit from Miltenyi Biotech (Miltenyi Biotech, 130-096-535, Bergisch Gladback, Germany) and corresponding protocol after which they were frozen and shipped to the MIMETAS research facility.

Barrier Integrity Assessment

After washing and medium addition to the gel channel and bottom perfusion channel, the top perfusion channel inlet and outlet were perfused with 40 µL and 30 µL of culture medium containing 0.5 mg/mL FITC-dextran (10kDa, Sigma, FD10S), respectively. Leakage of dextran from the lumen of the vessel into the adjacent ECM gel was imaged at 2-minute intervals using the Molecular Devices ImageXpress XLS Micro High-Content Imaging System (Molecular Devices, San Jose, CA, USA). The ratio between fluorescent signal in the top and middle channel was determined over time using FIJI (version 2 build 1.52d) and used to calculate apparent permeability (P_{app}) scores.

Flow Cytometry of Unstimulated and Stimulated T Cell Populations

Unstimulated and stimulated T cell populations were analyzed by flow cytometry after 48-hour culture in the absence or presence of CD3/CD28 Dynabeads to gain insight into the CD4+/CD8+ T cell ratio and their IFN-γ production. T cells were incubated (37°C, 5% CO2) with Brefeldin A (BioLegend, 420604, San Diego, CA, USA) (1:1000) for 4 hours before performing intracellular staining to inhibit protein transport processes. For both extracellular staining and intracellular staining, $1x10^5$ cells were transferred to a 96-well V-bottom plate (Greiner, M9686). The staining solution differs between extracellular and intracellular staining, the former using FACs buffer (Invitrogen, 00-4222-26, Waltham, MA, USA) and the latter permeabilization buffer (Invitrogen, 00-8333-56). Cells were washed twice in staining solution and stained for 30 minutes at 4°C in the dark. The following extracellular expression markers were used to stain the cells: CD14-PE/Cy7 (BioLegend, 3018813) (1:20), CD3-FITC (BioLegend, 300305) (1:20), CD4-PB (BioLegend, 317423) (1:20), CD8-PerCP (BioLegend, 344707) (1:20), LIVE/DEAD™ Fixable Aqua (Thermo Fisher Scientific, L34965) (1:1000) and Fc Receptor Binding Inhibitor (Thermo Fisher Scientific, 14-9161-73) (20 µl per test). For intracellular staining the following markers are used: IFN-γ-V450 (BD Biosciences, 560371) (1:20), LIVE/DEAD™ Fixable Aqua (Thermo Fisher Scientific, L34965) (1:1000) and Fc Receptor Binding Inhibitor (Thermo Fisher Scientific, 14-9161-73) (20 µl per test). Cells were stored in 200 µl FACs buffer at 4°C, until fluorescence was measured using BD FACSCanto™ II (BD Biosciences, Franklin Lakes, NJ, USA). Data analysis was performed using FlowJo™ v10.6.2 (Becton, Dickinson and Company, Franklin Lakes, NJ, USA).

Immunocytochemistry

Cultures in the OrganoPlate were fixed after the 48-hour co-culture period using 3.7% formaldehyde (Sigma, 252549) and permeabilized with 0.3% Triton X-100 (Sigma, T8787). Cultures were incubated with blocking solution containing 2% FCS, 2% serum albumin (BSA, Sigma, A2153), 0.1% Tween-20 (Sigma, P9416) for 45 min, washed with PBS containing 4% FCS and incubated with primary antibody (overnight) and secondary antibody (30 min.). The following primary antibodies were used to stain fixed cultures: Anti-CD3 1:100 (Abcam, ab5690, Cambridge, UK), anti-CD31 1:20 (Dako, M0823, Glostrup, Denmark), anti-ICAM-1 1:100 (R&D systems, BBA3, Minneapolis, MN, USA). The following secondary antibodies were used to stain fixed cultures: Goat anti-mouse IgG (H+L) Alexa Fluor PLUS 488 1:250 (Thermo Fischer Scientific, A32723), Goat anti-rabbit IgG (H+L) Alexa Fluor PLUS 555 1:250 (Thermo Fischer Scientific, A21428) and Goat anti-mouse IgG (H+L) Alexa Fluor 647 1:250 (Thermo Fischer Scientific, A21236). Hoechst 33342 (Thermo Fischer Scientific, H3570) was used as a nuclear stain. Steps were performed at room temperature. Z-planes were imaged using the Molecular Devices ImageXpress Micro Confocal High-Content Imaging System and analyzed using FIJI and IMARIS 9.6 trial version (Bitplane, Belfast, UK). Quantification of immunofluorescent ICAM-1 staining was performed by calculating the mean staining intensity of sum projections divided by the number of observed nuclei.

Supplementary figures

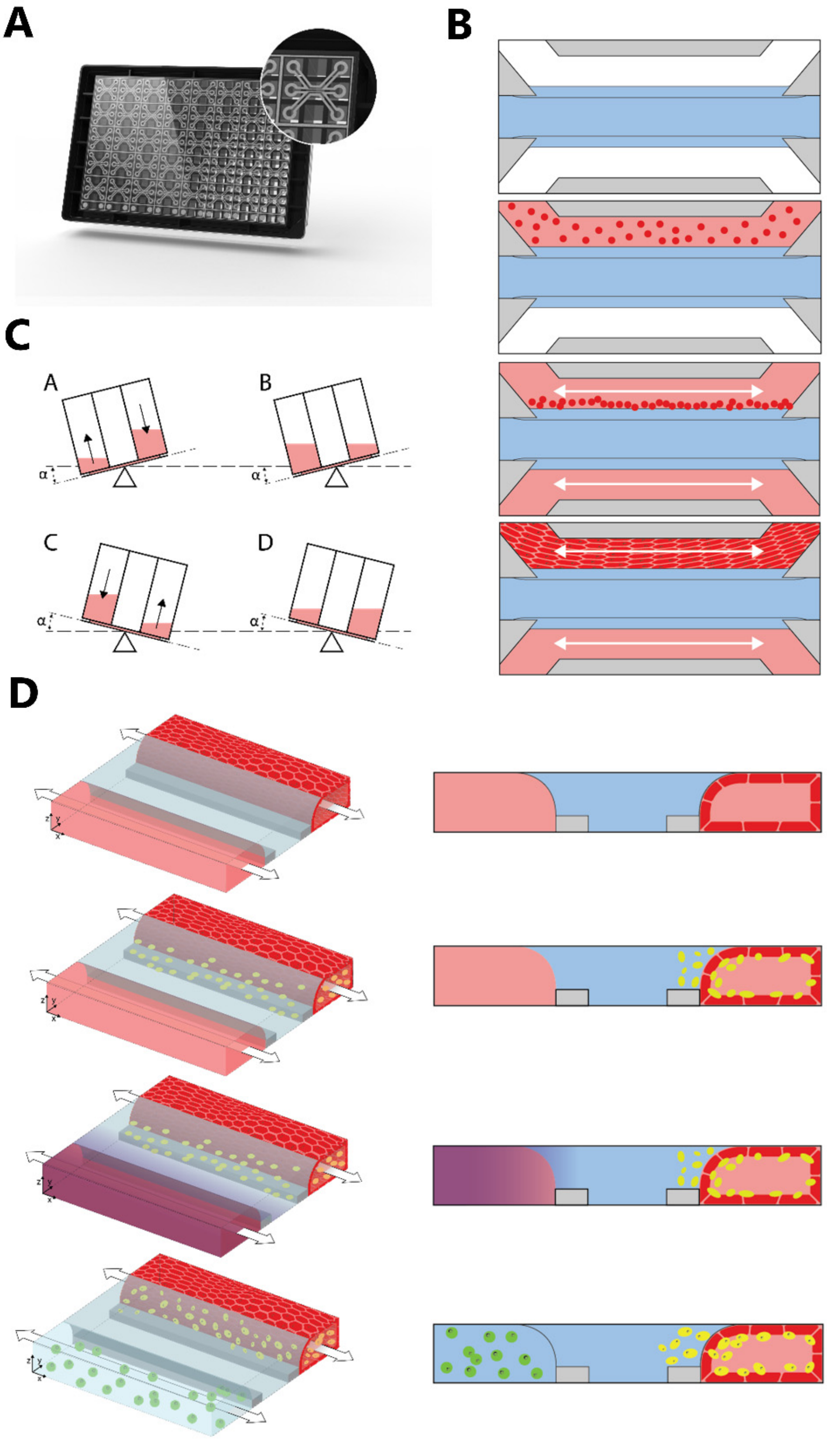

Figure S1. Schematic overviews of the microfluidic platform and co-culture model. (**A**) The OrganoPlate® 3-Lane is based on a 384-well microtiter plate and contains 40 individual microfluidic chips (inlay) that are integrated at the bottom. (**B**) A gel channel (blue) holds the extracellular matrix (ECM) in place through the PhaseGuide's pressure barrier function after which cells can be introduced in the top channel. Following sedimentation and attachment of cells to the ECM, the platform is placed on a tilted rocking platform (**C**) which results in gravity driven, continuous, and bi-directional perfusion of the cultures and the formation of a vessel with a clear apical and basal side (**D**). T cells (yellow) are apically added to the vessel and attach to the vessel wall after which they might undergo transendothelial migration (TEM). The extent to which TEM occurs can be modulated by the addition of a chemotactic gradient (purple hue) or the patterning of an additional cell type (green) in the bottom channel.

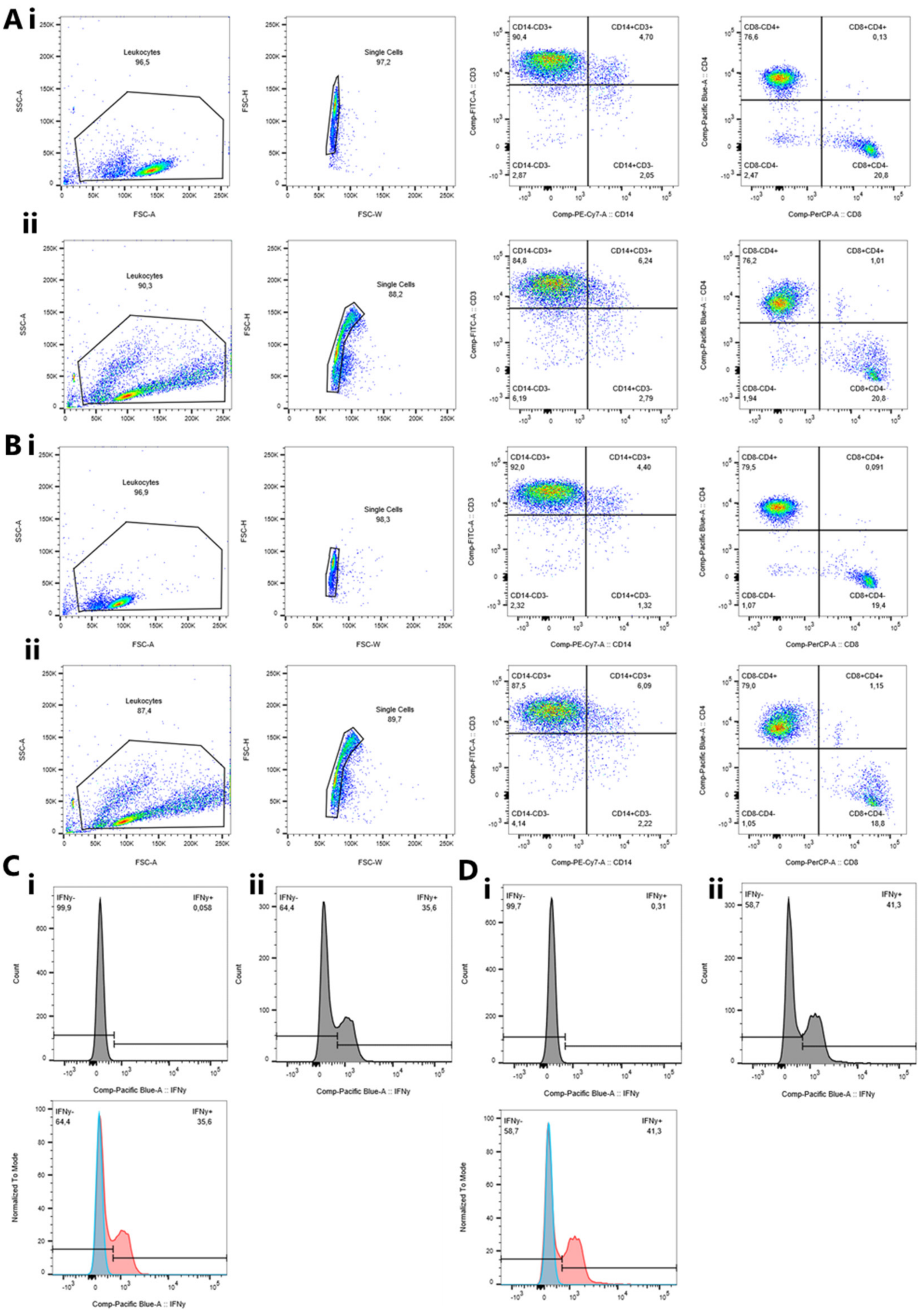

Figure S2. Characterization of unstimulated and (CD3/CD28) stimulated T cell populations. (**A** and **B**) Representative dot plots of T cell populations from 2 donors after isolation and 48-hour culture in the absence (unstimulated, i) or presence of (CD3/CD28) Dynabeads (Stimulated, ii). Live cells were stained using anti-CD3, anti-CD14, anti-CD4 and anti-CD8 as well as LIVE/DEAD™ Fixable Aqua stain. Analyses performed using FlowJo. (**C** and **D**) Representative histogram plot of interferon gamma (IFN-γ) production of T cells from 2 donors after 48-hour culture in the absence (unstimulated, i) or presence of (CD3/CD28) Dynabeads (Stimulated, ii). Cells were incubated with Brefeldin A for 4 hours before being fixed and stained using anti-IFN-γ and LIVE/DEAD™ Fixable Aqua stain. Analyses performed using FlowJo.

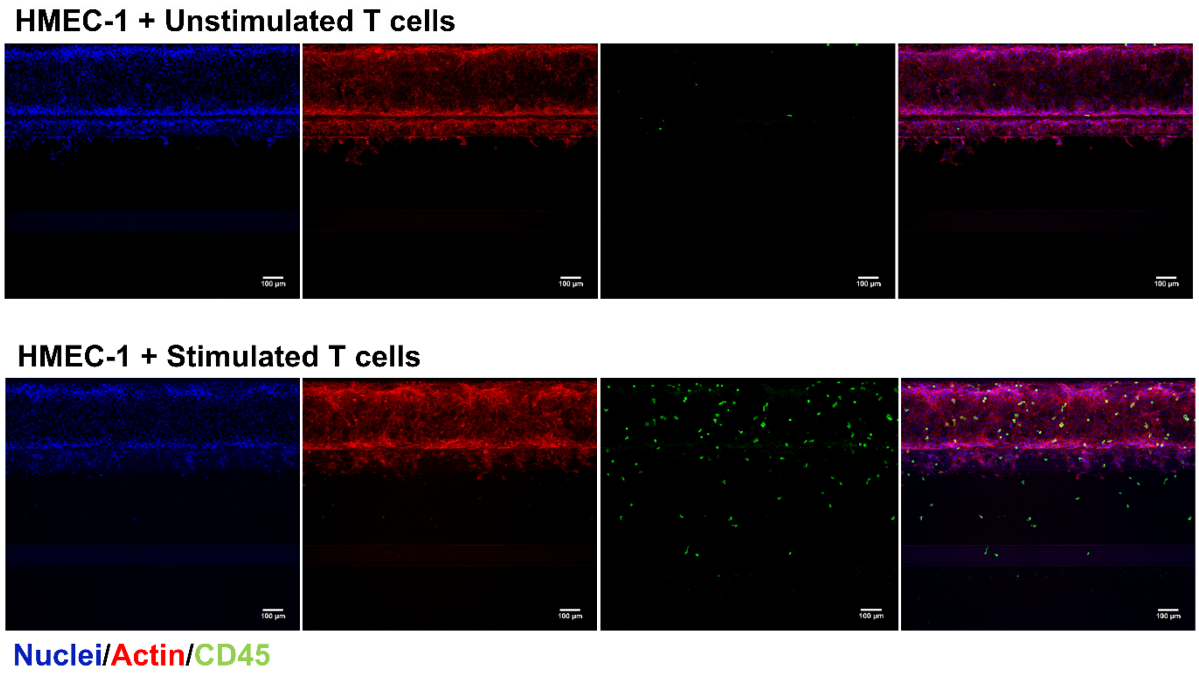

Figure S3. Immunofluorescent staining of a HMEC-1 vessel perfused with either unstimulated or (CD3/CD28) stimulated T cells. HMEC-1 vessels were co-cultured with T cells for 48 hours before being fixed using 3.7% formaldehyde. Cultures were stained for actin (red) and the immune cell marker CD45 (green). Nuclei are counterstained using Hoechst 33342. Scale bar = 100 µm.

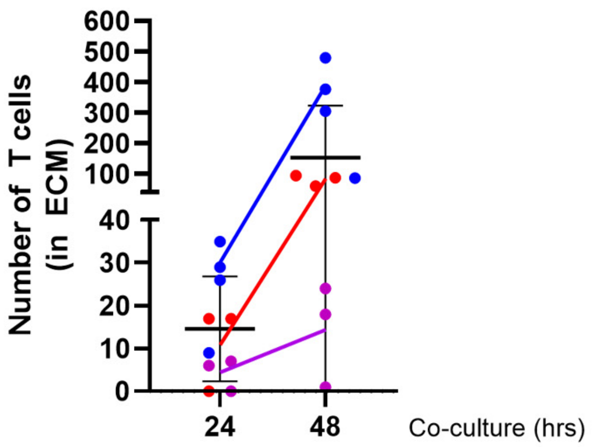

Figure S4. Migratory behavior of T cells varies between donors. Stimulated T cells isolated from different donors (blue, red and purple) show different migratory behavior in a co-culture with endothelial cells. Although already apparent after 24 hours, donor differences become more pronounced over time which results in an increased spreading of datapoints.

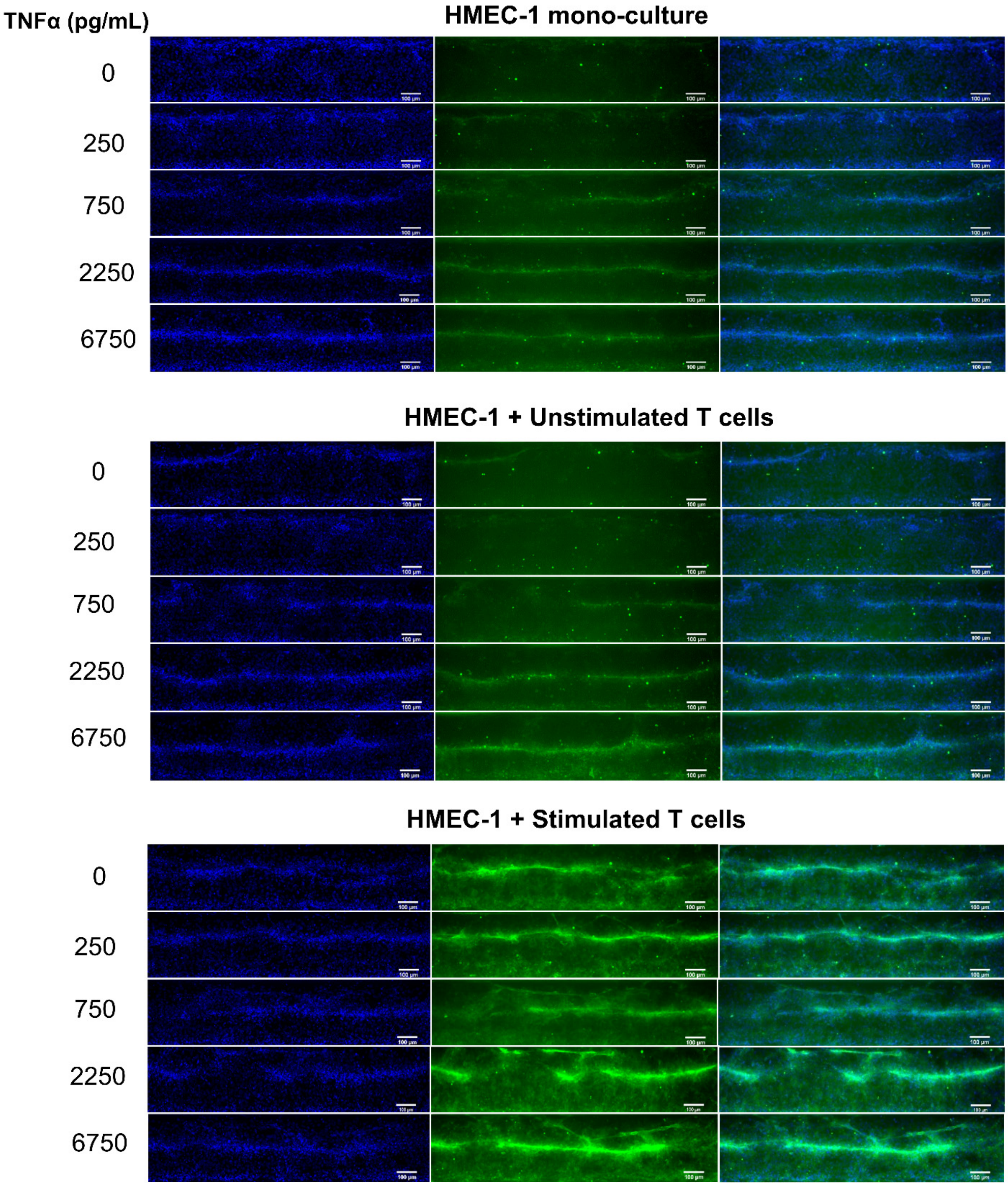

Figure S5. Expression of Intracellular Adhesion Molecule 1 (ICAM-1) increases upon TNF*α* pretreatment of HMEC-1 endothelial vessels. Immunofluorescent staining of HMEC-1 vessels after 16-hour treatment with TNF*α* (0 –6750 pg/mL), followed by 48 hours of either mono-culture or co-culture with T cells. Shown are maximum intensity projections for nuclei (blue) and sum intensity projections for ICAM-1 (green). Scale bar = 100 µm.

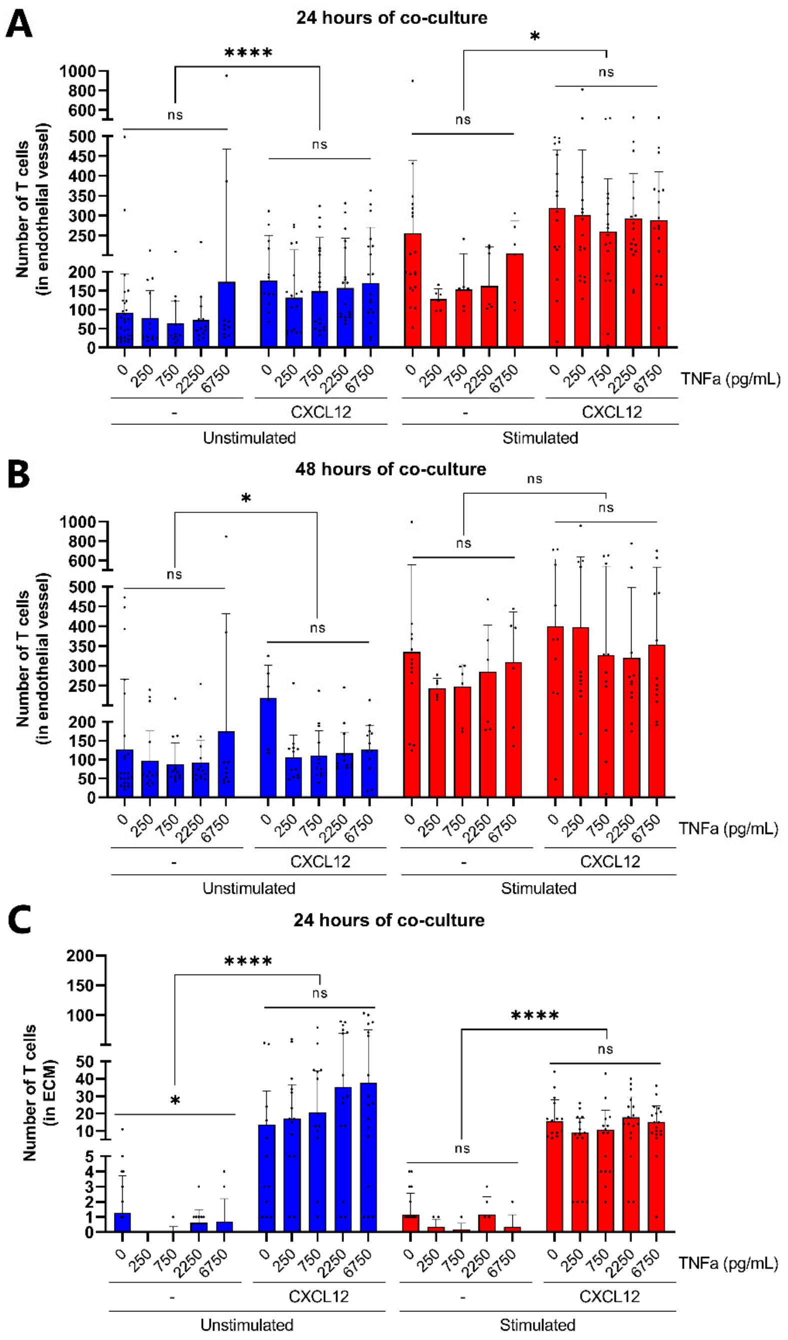

Figure S6. Inflammation potentiates migration of unstimulated T cells in response to CXCL12. (**A** and **B**) Quantification of T cell numbers in the endothelial vessel after 24 hours (**A**) 48 hours (**B**) of co-culture in response to TNFα pretreatment in the presence or absence of CXCL12. Shown are mean±SD and data points represent individual chips (N = 2–7, n = 3–5). Data was analyzed using One-way and Two-way ANOVA tests after log transformation, showing a significant effect of CXCL12 on the number of T cells in the endothelial vessel after TNFα pretreatment for both unstimulated (blue, **** $p < 0.0001$) and stimulated (red, * $p < 0.0219$) T cells at 24 hours as well as a significant effect at 48 hours for unstimulated T cells (*$p < 0.0381$). (**C**) Quantification of T cell numbers in the ECM compartment after 24 hours of co-culture in response to TNFα pretreatment in the presence or absence of CXCL12. Shown are mean±SD and data points represent individual chips (N = 2–7, n = 3–5). Data was analyzed using Two-way ANOVA test after log transformation, showing a significant effect of CXCL12 on the migration of T cells after TNFα pretreatment for both unstimulated (blue) and stimulated (red) T cells (**** $p < 0.0001$).

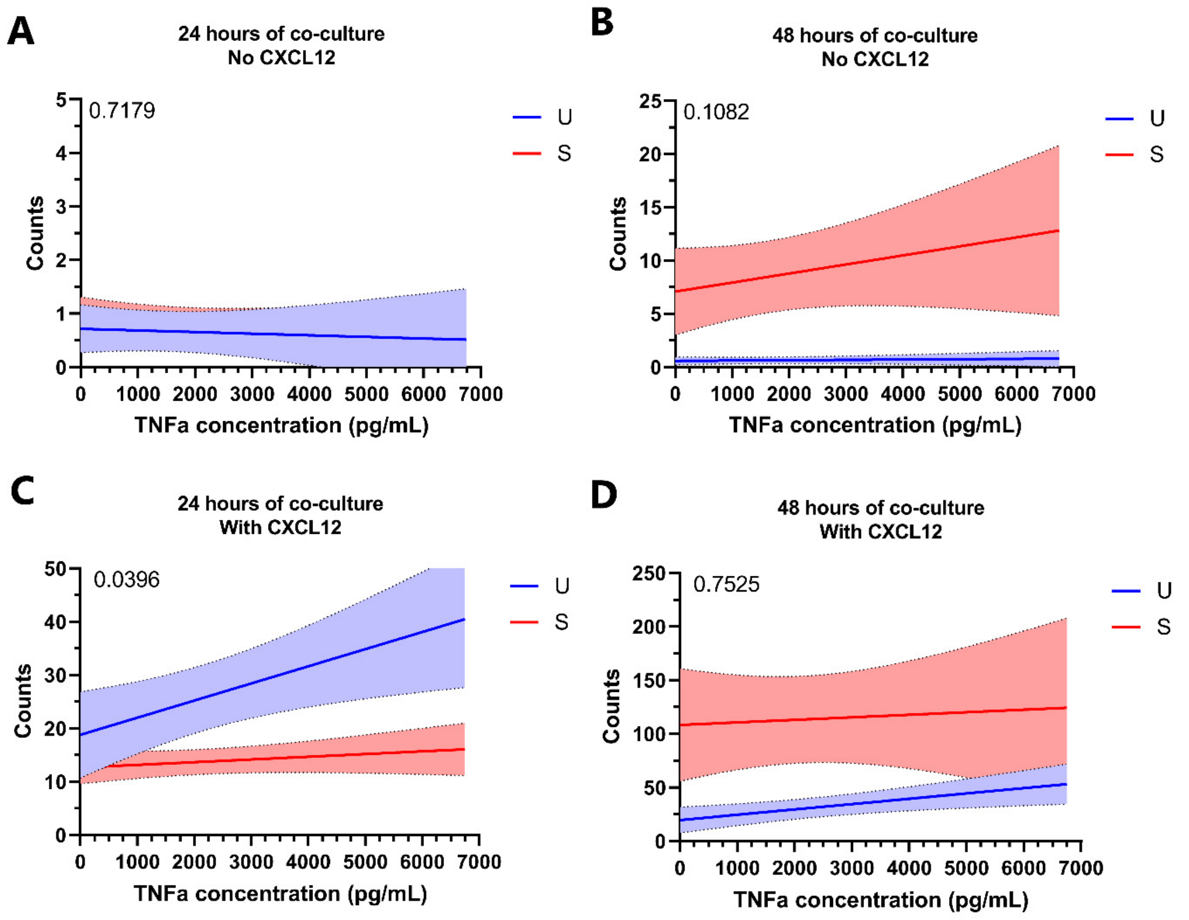

Figure S7. T cells respond differently to TNFα pretreatment depending on activation state and the presence or absence of CXCL12. HMEC-1 vessels were incubated with TNFα for 16 hours prior to addition of T cells to the apical side of the vessels. The number of migrating T cells in response to a concentration range of TNFα was analyzed using regression analysis, showing different responses for unstimulated (blue) and stimulated (red) T cells depending on the duration of co-culture and the presence of CXCL12. Shown are the fitted lines, the computed 95% confidence bands as well as p-values indicating similarity of regression curves.

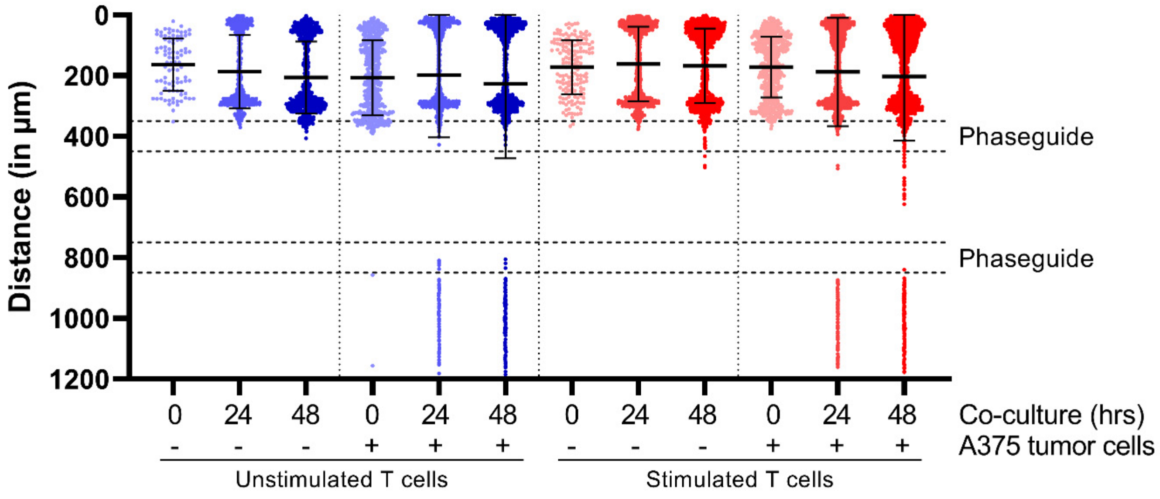

Figure S8. The migration of T cells is affected by the presence of A375 dermal melanoma cells. Unstimulated and stimulated T cells migrate towards A375 dermal melanoma cells. The positions of individual T cells throughout microfluidic chips were determined along the width of the chips, which measures 1200 µm in total. A microfluidic chip is subdivided in three compartments by the presence of two PhaseGuides, indicated by the dashed lines, and migration distance of T cells from the top of the chip was calculated and plotted.
